# Brahma‐Related Gene‐1 (BRG1) promotes the malignant phenotype of glioblastoma cells

**DOI:** 10.1111/jcmm.16330

**Published:** 2021-02-02

**Authors:** Yinan Wang, Chuan He Yang, Andrew P. Schultz, Michelle M. Sims, Duane D. Miller, Lawrence M. Pfeffer

**Affiliations:** ^1^ Department of Pathology and Laboratory Medicine (College of Medicine) and the Center for Cancer Research University of Tennessee Health Science Center Memphis TN USA; ^2^ Department of Pharmaceutical Sciences (College of Pharmacy) University of Tennessee Health Science Center Memphis TN USA

**Keywords:** BRG1, cell migration and invasion, gene expression, glioblastoma, STAT3

## Abstract

Glioblastoma multiforme (GBM) is an aggressive malignant brain tumour that is resistant to existing therapeutics. Identifying signalling pathways deregulated in GBM that can be targeted therapeutically is critical to improve the present dismal prognosis for GBM patients. In this report, we have identified that the BRG1 (Brahma‐Related Gene‐1) catalytic subunit of the SWI/SNF chromatin remodelling complex promotes the malignant phenotype of GBM cells. We found that BRG1 is ubiquitously expressed in tumour tissue from GBM patients, and high BRG1 expression levels are localized to specific brain tumour regions. Knockout (KO) of BRG1 by CRISPR‐Cas9 gene editing had minimal effects on GBM cell proliferation, but significantly inhibited GBM cell migration and invasion. BRG1‐KO also sensitized GBM cells to the anti‐proliferative effects of the anti‐cancer agent temozolomide (TMZ), which is used to treat GBM patients in the clinic, and selectively altered STAT3 tyrosine phosphorylation and gene expression. These results demonstrate that BRG‐1 promotes invasion and migration, and decreases chemotherapy sensitivity, indicating that it functions in an oncogenic manner in GBM cells. Taken together, our findings suggest that targeting BRG1 in GBM may have therapeutic benefit in the treatment of this deadly form of brain cancer.

## 1 INTRODUCTION

Gliomas are the most common primary intracranial neoplasms in adults and a leading cause of cancer‐related morbidity and mortality in the United States.^1^ While grade I glioma is the least malignant brain tumour, grade IV glioma (GBM, glioblastoma) is the most aggressive and deadliest of brain tumours. Surgical resection of GBM remains the primary treatment modality with present adjuvant chemotherapy and radiotherapy only providing slight improvement in the disease course and outcome.^2^ The overall median time for GBM recurrence after surgery is 7 months, and its 5‐year overall survival is dismal (<10% survival), which has remained relatively unchanged for decades.^1^

Chromatin regulation and epigenetically‐centred processes are tightly linked to cancer, and more than 20% of human cancers bear one or more mutations in the mammalian ATP‐dependent chromatin remodelling SWI/SNF complex. The SWI/SNF complex is an evolutionarily conserved multi‐subunit complex that is critical for gene regulation, differentiation, DNA repair and development. The two mutually exclusive catalytic subunits, BRM (Brahma/*SMARCA2*) and BRG1 (Brahma‐related gene 1/*SMARCA4*), utilize energy from ATP hydrolysis that is required to reposition and/or remodel nucleosomes at targeted loci, which opens or closes chromatin to regulate gene transcription.^3^, ^4^ BRG1 has been found to have tumour suppressing^5^, ^6^ and tumour promoting activity^7^, ^8^, ^9^ in a cancer context‐specific manner. In cancers of the lung, ovaries, skin and blood (lymphoma), BRG1 functions as a tumour suppressor with silencing or loss‐of‐function mutations being enriched.^6^, ^10^, ^11^ However, BRG1 mutations are relatively rare in GBM.^12^ In previous studies, we found BRG1 was highly expressed in the relatively quiescent subpopulation of cancer stem‐like cells (CSCs) isolated from GBM patient‐derived xenografts.^13^ Knockdown of BRG1 expression in GBM CSCs markedly increased cell proliferation and increased the expression of differentiation markers but reduced stem cell marker expression.^13^ These results indicated that BRG1 was critical for maintaining the stemness of GBM CSCs. In addition, BRG1 silencing in CSCs increased the tyrosine phosphorylation of the STAT3 transcription factor, which was basally high in these cells.

In the present study, we examined the functional consequences of knocking out BRG1 in relatively proliferative, non‐stem differentiated GBM cell lines by CRISPR‐CAS9 gene editing. In contrast to our previous findings that BRG1 shRNA knockdown increased the proliferation of GBM CSCs, knockout (KO) of BRG1 slightly reduced GBM cell proliferation. BRG1 KO also significantly inhibited GBM cell migration and invasion. In addition, BRG1‐KO sensitized GBM cells to the anti‐proliferative effects of the DNA alkylating agent temozolomide (TMZ), which is used in the frontline treatment of GBM patients. Furthermore, BRG1‐KO selectively increased the tyrosine phosphorylation of STAT3 and altered the expression of a number of STAT3‐regulated genes. Taken together, our results demonstrate that BRG‐1 promotes the malignant phenotype of GBM cells, by promoting invasion and migration, reguating the STAT3 pathway, and decreasing chemotherapy sensitivity. In addition, BRG1 has a context‐dependent role on the phenotypic behaviour of differentiated GBM cells and CSCs. Nonetheless, targeting BRG1 may have therapeutic benefit in the treatment of this deadly form of brain cancer by targeting both differentiated GBM tumour cells and CSCs.

## 2 MATERIALS AND METHODS

### 2.1 Biological reagents and cell cultures

Antibodies against the following proteins were procured from the respective vendors: BRG1 (Proteintech, Rosemont, IL); actin (Santa Cruz Biotechnology), pSTAT3, pSTAT1, STAT1 (Cell Signaling), and STAT3 (BD Biosciences). MT330 (Department of Neurosurgery, UTHSC) and LN229 (American Type Culture Collection) GBM cell lines were grown in DMEM containing 10% foetal bovine serum (Hyclone) supplemented with penicillin (100 IU/mL) and streptomycin (100 μg/mL) at 37°C with 5% CO_2_. The cells were authenticated by single‐tandem repeat analysis.

### 2.2 Gene expression analysis

Gene expression was determined in RNA isolated from de‐identified formalin‐fixed paraffin‐embedded (FFPE) patient biopsy specimens (UTHSC Tissue Services Core) as previously described [30]. In brief, total RNA was extracted using the QIAshredder and RNeasy mini kits (Qiagen Inc, Frederick, MD, USA). Quantitative real‐time PCR (qPCR) was performed using gene‐specific primers for BRG1 (forward 5’TACAAGGACAGCAGCAGTGG and reverse 3’TCCAGGTTGAAGGTCTGTGC).

TXNIP (forward 5’ATATGGGTGTGTAGACTACTGGG and reverse 3’GACATCCACCAGATCCACTACT), CXCL11 (forward 5’GACGCTGTCTTTGCATAGGC and reverse 3’ GGATTTAGGCATCGTTGTCCTTT), IL6 (forward 5’ ACTCACCTCTTCAGAACGAATTG and reverse 3’ CCATCTTTGGAAGGTTCAGGTTG), CDKN2A (forward 5’ GGGTTTTCGTGGTTCACATCC and reverse 3’ CTAGACGCTGGCTCCTCAGTA), IRF7 (forward 5’ GCTGGACGTGACCATCATGTA and reverse 3’ GGGCCGTATAGGAACGTGC), STAT3 (forward 5’and BETA‐ACTIN (forward 5’‐GGACTTCGAGCAAGAGATGG‐3’ and reverse 5’‐AGCACTGTGTTGGCGTACAG‐3’) with an iScript one‐step RT‐PCR kit containing SYBR Green (Bio‐Rad, Hercules, CA, USA). The reaction parameters were as follows: cDNA synthesis at 50°C for 20 minutes, transcriptase inactivation at 95°C for 5 minutes, and PCR cycling at 95°C for 10 seconds and 60°C for 30 seconds for 40 cycles. Gene expression was normalized relative to ACTIN expression.

### 2.3 RNA‐ISH

For RNA‐in situ hybridization (ISH) on FFPE tissue, 5‐μm sections on glass slides were baked for 1 hour at 60°C, deparaffinized and treated for target retrieval according to the manufacturer's protocol. The FFPE sections were then incubated with RNAscope ISH probes (Advanced Cell Diagnostics) and hybridized to visualize target RNA signals, according to the RNAscope Fluorescent Kit user manual. Slides were counterstained with DAPI (4′,6‐diamidino‐2‐phenylindole) to stain nuclei. Images were captured on a laser‐scanning confocal microscope (Zeiss model LSM700).

### 2.4 Immunoblot analysis

Total cell lysates (25 μg) were separated by SDS‐PAGE, immunoblotted with the indicated antibodies and visualized as previously described.^14^

### 2.5 Bioinformatic analysis

Several cancer genomic data sets for GBM patients^15^, ^16^, ^17^ were queried for epidermal growth factor receptor (EGFR), phosphatase and tensin homolog (PTEN) and SMARCA4/BRG1 mutations, deletions and amplifications using the cBioPortal tool.^18^ Gene expression data in The Cancer Genome Atlas (TCGA) for all normal and GBM samples were analysed using the GlioVis data portal tool.^19^ Statistical analyses were performed using Graphpad Prism (GraphPad software).

### 2.6 Generation of BRG1‐KO cells

The lentiviral CRISPR/Cas9‐mediated BRG1 knockout vectors were constructed by cloning three BRG1 guide RNAs (gRNA1: 5’‐ GGAGTTCCGCCCAGGGG ‐3’; gRNA2: 5’‐GGCCTGCTGTMTTTGG‐3’; and gRNA3: 5’‐ TGCAGTGGCACCATGGGCGC ‐3’) into the Bsm I site of lentiviral vector pLenti CRISPR V2. A control vector was constructed by inserting the EGFP gRNA sequence into the lentiviral vector. Lentivirus were produced by packaging in 293FT cells as we published previously.^20^ Stable pools of BRG1‐KO cells were generated by transducing GBM cells with the lentiviral CRIPSR/Cas9 vectors and selected with 3 μg/mL puromycin. Puromycin‐resistant cells were expanded in the absence of puromycin and subsequently used in experiments.

### 2.7 Cell proliferation, invasion and wound healing

For cell proliferation analysis, cells were plated into 96 well plates (5 × 10^3^ cells/well) and after 24 hours placed in the Incucyte live cell analysis system (Essen Bioscience) and treated in the presence or absence of TMZ (0, 10, 20 and 40 μmol/L) in a 37°C incubator, and cell numbers enumerated with the manufacturer provided software tools. Invasion assays through Matrigel using transwell inserts (BD Biosciences) were performed as previously described.^21^ Confluent cell monolayers were wounded with a sterile 1000 µL pipette tip, and phase‐contrast images were recorded to assess the extent of wound healing. At least 3 fields were examined for each experimental condition.

### 2.8 Statistical analyses

At least two independent experiments were performed in duplicate, and data are presented as means ± standard deviation (SD). Analysis of Variance (ANOVA) and post hoc least significant difference analysis or Student's *t* tests were performed. *P* values < .05 (*), .01 (**) and .001 (***) were considered statistically significant.

## 3 RESULTS

### 3.1 BRG1 is highly expressed in GBM tumour tissue

**FIGURE 1:**
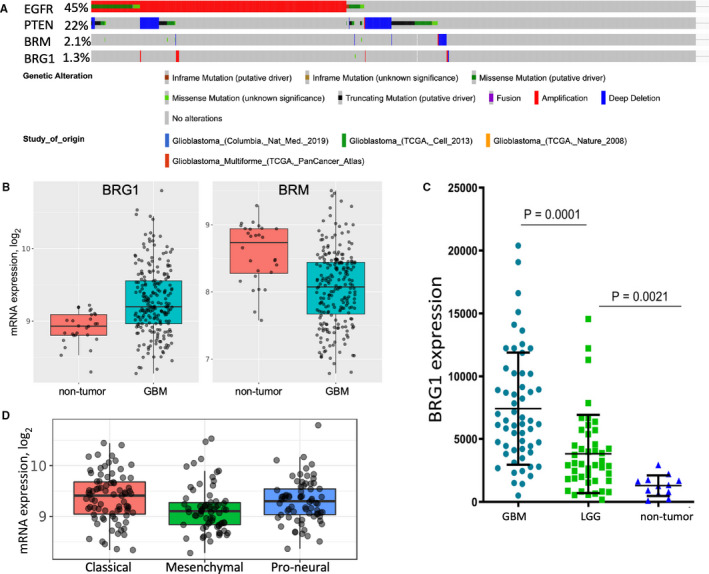
BRG1 gene alterations and expression in brain cancer patient samples. (A) Large‐scale cancer genomic data sets for GBM patients were queried for EGFR, PTEN, SMARCA2/BRM and SMARCA4/BRG1 mutations, deletions and amplifications using the cBioPortal tool. (B) Non‐tumour tissue (10) and GBM patient samples (528) in the TCGA database were compared for BRG1 and BRM expression. (C) RNA was extracted from de‐identified formalin‐fixed paraffin‐embedded patient biopsies identified as GBM, LGG or normal brain tissue, and BRG1 expression was determined by qPCR (n = 3), and normalized to actin expression. (D) BRG1 expression in the different GBM molecular subtypes in the REMBRANDT database

Mutation and deletion of BRG1/SMARCA4 have been shown to contribute to a range of human malignancies.^12^, ^22^ Previous studies have established that mutations in EGFR result in its overexpression, while genomic mutations in PTEN tumour suppressor leads to its down‐regulation in GBM.^23^ Consistent with previous findings, analysis of cancer genomics data in several GBM patient databases using the cBioPortal tool^18^ showed that EGFR amplification (46%) and PTEN deletion (25%) frequently occur in GBM (Figure 1A). In contrast, the BRG1 and BRM genes are rarely altered at the genetic level in GBM (<2%). BRG1 and BRM gene expression in TCGA was then analysed in non‐tumour brain tissue and GBM tumour tissue using the GlioVis portal.^19^ We found that BRG1 was expressed at significantly higher levels in GBM tumour tissue as compared to non‐tumour brain tissue, while BRM was expressed at lower levels in tumor tissue (Figure 1B). To independently validate that BRG1 was overexpressed in GBM, we extracted RNA from FFPE tissue blocks of non‐tumour tissue, low‐grade glioma (LGG) and GBM patients from the UTHSC tissue archive and determined BRG1 expression by qPCR. BRG1 expression was found to be significantly higher in GBM as compared to LGG, and the lowest BRG1 expression was found in non‐tumour brain tissue (Figure 1C). In addition, BRG1 is expressed in all three molecular subtypes of GBM with highest expression in the classical subtype and lowest in the mesenchymal subtype (Figure 1D). Taken together, these data are consistent with the basic hypothesis that BRG1 plays a pro‐tumorigenic role in GBM.

**FIGURE 2:**
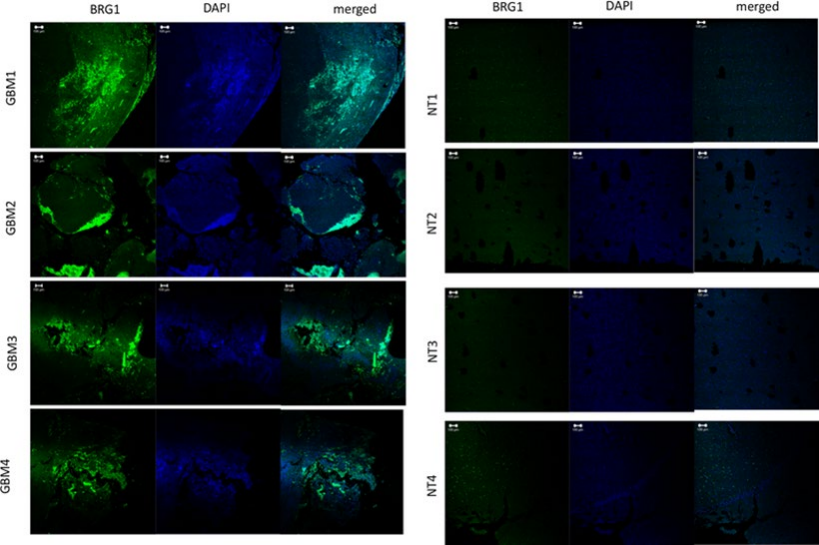
BRG1 expression at the mRNA level in GBM tumour tissue. Slides were prepared from GBM tumour tissue and non‐tumour tissue (NT), subjected to RNA‐ISH using the RNAscope technology and images analysed by confocal microscopy (20 × tile‐scan magnification). RNA‐ISH was performed with the gene probes for BRG1 (green), and nuclei were DAPI counterstained (blue)

To further characterize BRG1 gene expression at the cellular level in human tissues, we performed RNA‐ISH on sections cut from FFPE tissue blocks from GBM patient specimens and non‐tumour brain tissue, as described previously.^24^ As shown in Figure 2, cells expressing high levels of BRG1 RNA (green dots) as detected by the RNA‐ISH probe were found localized to discrete regions of the tumours in four different GBM patients. In contrast, BRG1 was expressed at relatively low levels throughout non‐tumour brain tissue as compared to GBM tumours without any areas of high BRG1 expression observed. It is important to note that there were also areas with relatively low BRG1 gene expression in GBM patient tissue (Figure 2).

### 3.2 Knockout of BRG1 in GBM cells selectively promotes tyrosine phosphorylation of STAT3, but not of STAT1

**FIGURE 3:**
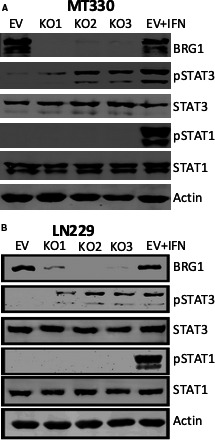
The effect of BRG1‐KO on the tyrosine phosphorylation of STAT3 in GBM cells. BRG1 was knocked out in MT330 (A) and LN229 (B) cells by CRISPR‐Cas9 gene editing with three different gRNAs and cell pools were isolated after puromycin selection. As a control, cells were transduced with empty lentiviral vector (EV). Cell lysates were analysed by immunoblotting for BRG1, pY705‐STAT3, pY701‐STAT1, total STAT1 and STAT3, and actin. As a positive control for STAT1 and STAT3 tyrosine phosphorylation, cell lysates were prepared from EV cells that were treated with IFN‐con1 for 30 min

To define the functional significance of the expression of BRG1 in differentiated GBM cells grown in vitro, we used CRISPR/Cas9 lentivirus encoding three different gRNAs to delete the BRG1 gene from LN229 and MT330 GBM cells. As a control in these studies, cells were transduced with empty vector (EV). After puromycin selection, the individual pools of cells transduced with each gRNA were grown and maintained in the absence of puromycin. To validate that BRG1 was indeed knocked out at the protein level, lysates of EV and three individual BRG1‐KO MT330 cell lines were prepared, fractionated on SDS‐PAGE, and immunoblotted for BRG1. As expected, BRG1 is clearly expressed in both control (EV) MT330 and LN229 cells (Figure 3), but BRG1 expression is undetectable in BRG‐1KO cell lines. BRG1 knockdown by shRNA in GSCs isolated from several patient‐derived xenografts was previously found to increase STAT3 tyrosine 705 phosphorylation (pTyr‐STAT3).^13^ STAT1 and STAT3 are constitutively tyrosine phosphorylated in GBM cells, and both of these activated STAT proteins have been shown to promote GBM tumorigenesis.^25^, ^26^ While total STAT3 levels were unaffected, three individual BRG1‐KO MT330 and LN229 cell lines developed using different gRNAs showed a dramatic increase in STAT3 tyrosine phosphorylation (Figure 3). Furthermore, the effect on tyrosine phosphorylation was selective for STAT3 since tyrosine phosphorylation of STAT1 was not increased in either MT330 and LN229 cells. As a positive control, EV MT330 cells were treated with the cytokine interferon (IFN), which induced tyrosine phosphorylation of both STAT3 and STAT1 as described previously.^27^ This result suggests that the effect of BRG1 on pTyr‐STAT3 was distinct from the IFN signalling pathway. It is noteworthy that the basal level of pTyr‐STAT3 in BRG1‐KO MT330 and LN229 cells was similar to that induced by IFN treatment of EV MT330 and LN229 cells.

### 3.3 Knockout of BRG1 in GBM cells reduces cell migration and invasion, and increases sensitivity to the anti‐proliferative effect of temozolomide

**FIGURE 4:**
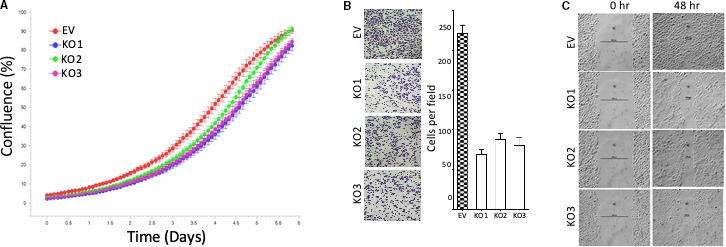
The effect of BRG1‐KO on GBM cell proliferation, invasion and migration. (A) EV and BRG1‐KO MT330 GBM cells were plated into 96 well plates, and cell proliferation determined by live cell analysis. (B) Transwell plate invasion assays on EV and BRG1‐KO MT330, and invading cells were stained with crystal violet and quantified. (C) Wound healing assays on EV and BRG1‐KO MT330 cells

To further characterize the functional consequences of BRG1 deletion, we then assessed how BRG1 deletion affects GBM cell proliferation, migration and invasion. While knockout of BRG1 expression in MT330 cells only slightly inhibited GBM cell proliferation as determined by Incucyte live cell analysis (Figure 4A), BRG1‐KO significantly reduced cell invasion in Matrigel transwell assays (Figure 4B) and cell migration in wound healing assays (Figure 4C).

**FIGURE 5:**
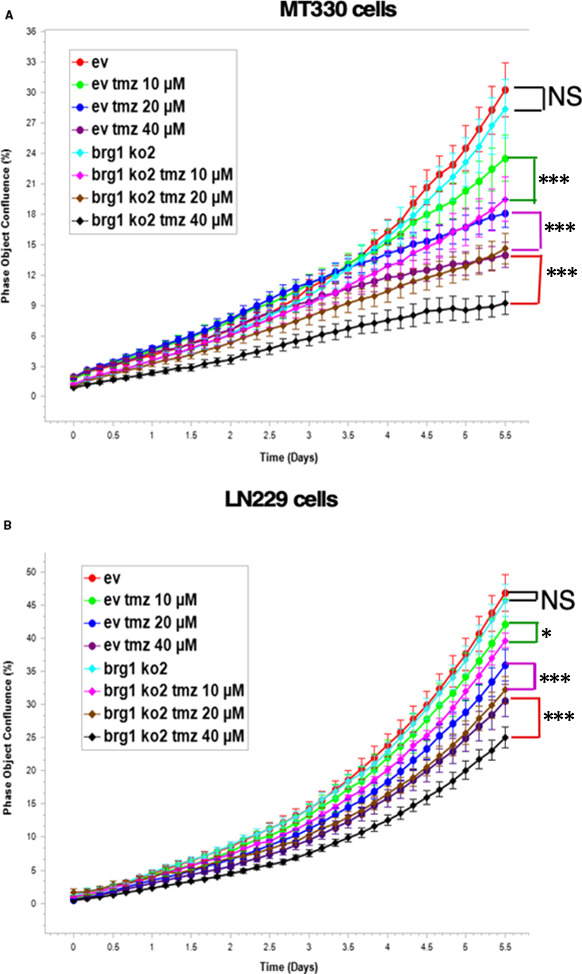
BRG1‐KO sensitizes GBM cells to temozolomide. EV and BRG1‐KO (A) MT330 and (B) LN229 GBM cells were treated with TMZ (0, 10, 20 and 40 μM), and cell proliferation determined by live cell analysis

Consistent with previous findings,^28^ treatment of both MT330 and LN229 cells with TMZ resulted in a dose‐dependent reduction in GBM cell proliferation. Most interestingly, the anti‐proliferative effect of TMZ was increased in both MT330 and LN229 BRG1‐KO cells at each dose of TMZ tested as compared to control EV cells (Figure 5). The sensitizing effect of BRG1‐KO to TMZ was more pronounced in MT330 cells than in LN229 cells.

### 3.4 BRG1‐KO regulates gene expression in GBM cells

**FIGURE 6:**
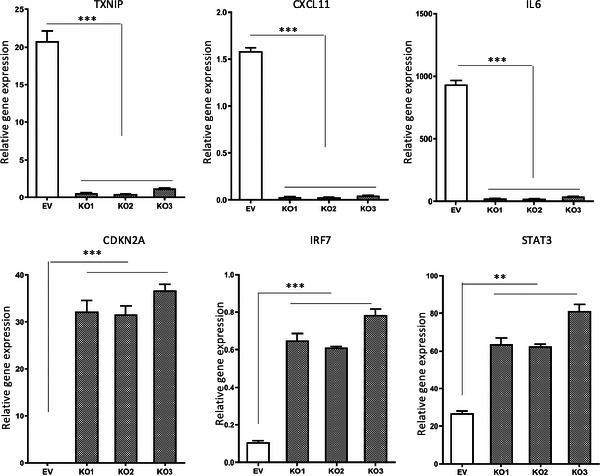
FIGUREThe effects of BRG1‐KO on expression of STAT3 target genes. RNA was prepared from control (EV) and BRG1‐KO MT330 cells, and the expression of TXNIP, CXCL11, IL6, CDKN2A, and IRF7 was determined by qPCR

Being a subunit of a chromatin remodelling complex, BRG1 plays a critical role in regulating gene transcription.^3^, ^4^ By microarray analysis of gene expression, we previously found that knockdown of BRG1 in GSCs selectively up‐regulated and down‐regulated the expression of a specific subset of genes, including several IFN stimulated genes (ISGs).^13^ To determine the effect of BRG1‐KO in established GBM cells on the expression of this subset of genes, we isolated RNA from EV and BRG1‐KO MT330 cells and analysed expression of the following ISGs that are known to play distinct roles in tumorigenesis: TXNIP, CXCL11, IL6, CDKN2A and IRF7. While expression of TXNIP, CXCL11 and IL6 was markedly down‐regulated in BRG1‐KO MT330 cells as compared to control (EV) cells, the expression of CDKN2A and IRF7 was significantly up‐regulated in BRG1‐KO cells (Figure 6). Interestingly, BRG1‐KO also up‐regulated the expression of STAT3. These results indicate that BRG1 promotes the expression of TXNIP, CXCL11 and IL6, while suppresses the expression of CDKN2A and IRF7.

## 4 DISCUSSION

The SWI/SNF chromatin remodelling complex regulates gene expression by binding to the promoters and enhancers of genes across the genome.^29^, ^30^, ^31^, ^32^ This complex plays critical roles during embryonic development and regulates stem cell renewal and differentiation.^33^, ^34^ Mutation and deletion of BRG1 have been shown to contribute to a range of human malignancies.^12^, ^22^ In the present study, we found that the BRG1 gene is rarely altered at the genetic level in GBM (<2%). In contrast, EGFR amplification (46%) and PTEN deletion (25%) are known to frequently occur. Furthermore, we found that BRG1 expression was highest in GBM tumour tissue as compared to non‐tumour tissue, and intermediate levels of BRG1 were present in low‐grade glioma. BRG1 was highly expressed in all three molecular subtypes with highest expression in the classical GBM subtype. Furthermore, high BRG1 expression was found to be selectively localized to distinct regions on GBM tumour specimens as determined by RNA‐ISH. Taken together, these findings lead us to suggest that BRG1 may play a pro‐tumorigenic role in GBM. An important goal of personalized medicine is to employ gene sequencing to identify targetable mutations for which there may be an effective drug. Our findings suggest that BRG1 is not mutated and is highly expressed in many patients with GBM. Thus, BRG1 may be an attractive druggable target in GBM.

In some forms of cancer, BRG1 is associated with tumour progression, while in others, it is associated with tumour suppression.^35^, ^36^, ^37^, ^38^, ^39^ Since BRG1 is highly expressed in differentiated GBM cells, a goal of the present study was to define the cellular function of BRG1 in GBM. We employed the approach of knocking out BRG1 expression in two different GBM cell lines by CRISPR‐CAS9 gene editing using three different guide RNAs and then isolated pools of KO cells. After validating BRG1‐KO by immunoblotting, we found that loss of BRG1 expression in both MT330 and LN229 cells resulted in high basal levels of tyrosine phosphorylation of the STAT3 transcription factor, but phosphorylation of STAT1 was unaffected. The effects of BRG1 on STAT phosphorylation were selective for STAT3, which is consistent with previous findings that silencing BRG1 with shRNA in GBM cancer stem cells resulted in high STAT3 tyrosine phosphorylation but not in the phosphorylation of other STAT proteins.^13^ We then determined the effect of BRG1‐KO on the expression of a number of known STAT3‐activated genes.^13^, ^26^ BRG1‐KO down‐regulated the expression of TXNIP, CXCL11 and IL6, indicating that BRG1 promotes the expression of TXNIP, CXCL11 and IL6. TXNIP blocks glucose uptake and restricts aerobic glycolysis, as well is involved in redox regulation.^40^, ^41^, ^42^, ^43^, ^44^ IL6 and CXCL11 are cytokines and chemokines that promote immune evasion in GBM.^45^, ^46^ Taken together, BRG1 may promote GBM tumorigenesis by increasing the expression of genes involved in immune evasion and metabolic reprogramming in GBM. Furthermore, it is particular interest that IL6 also activates STAT3 tyrosine phosphorylation, indicating that the BRG1/IL6/STAT3 positive feedback loop may promote GBM tumorigenesis. In agreement with the present findings, BRG1 was found to promote TXNIP, CXCL11 and IL6 expression in GBM cancer stem cells,^13^ suggesting a broad role of these genes in the pro‐tumorigenic role of BRG1 in GBM.

In addition, we found that BRG1‐KO up‐regulated expression of CDKN2A and IRF7, indicating that BRG1 suppresses their expression. Deletion of *CDKN2A* gene or silencing of its expression by promoter methylation is prevalent in several human cancers, consistent with CDKN2A being a tumour suppressor gene. Furthermore, IRF7 promotes immune surveillance by natural killer and T cells.^47^ Taken together, BRG1 may promote GBM tumorigenesis by down‐regulating the expression of tumour suppressor genes and gene involved in immune surveillance in GBM. Consistent with the present findings, BRG1 was found to suppress CDKN2A and IRF7 expression in GBM cancer stem cells.^13^

Somewhat contradictory findings have been reported on the role of BRG1 on GBM cell proliferation. Knockdown of BRG1 in embryonic stem cells and in GBM CSCs was reported to reduce cell proliferation.^48^, ^49^, ^50^ However, we previously showed that BRG1 knockdown in GBM CSCs increased cell proliferation in vitro and in vivo.^13^ BRG1 loss also has been shown to increase the proliferation of various cancer cell lines and embryonic stem cells.^37^, ^51^, ^52^, ^53^, ^54^ In the present study, we found that BRG1‐KO had only a minor inhibitory effect on the proliferation of both MT330 and LN229 GBM cell lines. This finding is in stark contrast to the marked increase in the proliferation of GBM CSCs upon BRG1 knockdown.^13^ Taken together, these results suggest that, while BRG1 plays an important role in suppressing the proliferation of GBM CSCs, BRG1 plays a relatively minor role in the proliferation of differentiated GBM cells. However, in the present study we found that BRG1 plays an important role in GBM cell migration and invasion. In both MT330 and LN229 cells, BRG1‐KO markedly reduced cell migration in wound healing and invasion through Matrigel in transwell assays, indicating that BRG1 promotes GBM cell migration and invasion, which are typical characteristics of GBM in vivo and provides further support for a pro‐tumorigenic role of BRG1 in GBM.

Another important finding from the present study is that although BRG1 does not have a major role in GBM cell proliferation, BRG1 appears to play an important role in promoting the resistance of GBM cells to the DNA alkylating TMZ, which is a frontline chemotherapy used on GBM patients following tumour resection. Since inherent and acquired resistance of patients to TMZ therapy is an important clinical problem, our finding that BRG1‐KO sensitizes GBM cells to the anti‐proliferative effect of TMZ is highly significant. Consistent with our findings that BRG1‐KO increases TMZ sensitivity of GBM cell lines, BRG1 knockdown in GBM CSCs also increased TMZ sensitivity in vitro. BRG1 knockdown also increased the proliferation of GBM CSCs, which may increase chemosensitivity, because CSCs proliferate relatively slowly, and chemotherapeutic drugs target proliferating cancer cells.^55^, ^56^ However, non‐stem, differentiated GBM cell lines already proliferate rather rapidly and BRG1‐KO had no significant effect on their proliferation but did increase chemosensitivity. These findings suggesti that BRG1 may increase the sensitivity of non‐stem GBM cells to TMZ through a mechanism independent of its effects on cell proliferation. The BRG1‐containing SWI/SNF chromatin remodelling complex regulates various DNA repair pathways, including DNA double‐strand breaks by enabling homologous recombination repair, and BRG1 loss may make cells susceptible to cisplatin and UV radiation.^57^, ^58^

In summary, we demonstrate that BRG1 is highly expressed in GBM tumour tissue and in non‐stem GBM cell lines. Although BRG1‐KO had little effect on GBM cell proliferation, it significantly inhibited GBM cell migration and invasion. In addition, BRG1‐KO sensitized GBM cells to TMZ, which is used to treat GBM patients. BRG1 selectively suppressed the constitutive tyrosine phosphorylation of STAT3 transcription factor. BRG1 promoted the expression of STAT3‐regulated genes involved in immune evasion and metabolic reprogramming in GBM, and BRG1 suppressed the expression of tumour suppressor genes and genes involved in immune surveillance in GBM. How BRG1 mediates these effects in proliferating GBMs, through its chromatin modifying activity or are their other interactions, remains to be established. Taken together, our results demonstrate that BRG1 promotes the malignant phenotype of GBM cells. Thus, BRG1 may be an attractive new target in this most deadly form of brain cancer.

## CONFLICT OF INTEREST

All authors declare no potential conflicts of interest.

## AUTHOR CONTRIBUTION

**Yinan Wang:** Data curation (equal); Investigation (equal); Methodology (equal); Resources (equal); Validation (equal); Writing‐original draft (supporting); Writing‐review & editing (supporting). **Chuan He Yang:** Conceptualization (supporting); Data curation (supporting); Formal analysis (supporting); Investigation (equal); Methodology (equal); Resources (equal); Software (equal); Supervision (equal); Writing‐original draft (supporting); Writing‐review & editing (supporting). **Andrew Schultz:** Data curation (equal); Investigation (equal); Visualization (supporting); Writing‐review & editing (supporting). **Michelle Sims:** Data curation (equal); Investigation (equal); Methodology (equal); Resources (equal); Visualization (supporting); Writing‐original draft (supporting); Writing‐review & editing (supporting). **Duane Miller:** Conceptualization (supporting); Writing‐original draft (supporting); Writing‐review & editing (supporting). **Lawrence Pfeffer:** Conceptualization (lead); Formal analysis (lead); Funding acquisition (lead); Project administration (equal); Supervision (lead); Visualization (lead); Writing‐original draft (lead); Writing‐review & editing (lead).

## Data Availability

The data that support the findings of this study are available from the corresponding author upon reasonable request.

## References

[1] Surawicz TS , Davis F , Freels S , Laws ER Jr , Menck HR . Brain tumor survival: results from the National Cancer Data Base. J Neurooncol. 1998;40(2):151‐160. https://doi.org10.1023/a:1006091608586. 9892097 10.1023/a:1006091608586

[2] Stupp R , Mason WP , van den Bent MJ , et al. Radiotherapy plus concomitant and adjuvant temozolomide for glioblastoma. N Engl J Med. 2005;352(10):987‐996. https://doi.org10.1056/NEJMoa043330. 15758009 10.1056/NEJMoa043330

[3] Trotter KW , Archer TK . The BRG1 transcriptional coregulator. Nucl Recept Signal. 2008;6:e004. https://doi.org10.1621/nrs.06004 10.1621/nrs.06004 PMC2254329 18301784

[4] Tolstorukov MY , Sansam CG , Lu P , et al. Swi/Snf chromatin remodeling/tumor suppressor complex establishes nucleosome occupancy at target promoters. Proc Natl Acad Sci USA. 2013;110(25):10165‐10170. https://doi.org10.1073/pnas.1302209110 23723349 10.1073/pnas.1302209110 PMC3690861

[5] Kadoch C , Hargreaves DC , Hodges C , et al. Proteomic and bioinformatic analysis of mammalian SWI/SNF complexes identifies extensive roles in human malignancy. Nat Genet. 2013;45(6):592‐601. https://doi.org10.1038/ng.2628. 23644491 10.1038/ng.2628 PMC3667980

[6] Shain AH , Pollack JR . The spectrum of SWI/SNF mutations, ubiquitous in human cancers. PLoS One. 2013;8(1):e55119. https://doi.org10.1371/journal.pone.0055119. 23355908 10.1371/journal.pone.0055119 PMC3552954

[7] Sentani K , Oue N , Kondo H , et al. Increased expression but not genetic alteration of BRG1, a component of the SWI/SNF complex, is associated with the advanced stage of human gastric carcinomas. Pathobiology. 2001;69(6):315‐320. https://doi.org10.1159/000064638. 12324708 10.1159/000064638

[8] Sun A , Tawfik O , Gayed B , et al. Aberrant expression of SWI/SNF catalytic subunits BRG1/BRM is associated with tumor development and increased invasiveness in prostate cancers. Prostate. 2007;67(2):203‐213. https://doi.org10.1002/pros.20521. 17075831 10.1002/pros.20521

[9] Guerrero‐Martinez JA , Reyes JC . High expression of SMARCA4 or SMARCA2 is frequently associated with an opposite prognosis in cancer. Sci Rep. 2018;8(1):2043. https://doi.org10.1038/s41598‐018‐20217‐3 29391527 10.1038/s41598-018-20217-3 PMC5794756

[10] Imielinski M , Berger AH , Hammerman PS , et al. Mapping the hallmarks of lung adenocarcinoma with massively parallel sequencing. Cell. 2012;150(6):1107‐1120. https://doi.org10.1016/j.cell.2012.08.029. 22980975 10.1016/j.cell.2012.08.029 PMC3557932

[11] Le Loarer F , Watson S , Pierron G , et al. SMARCA4 inactivation defines a group of undifferentiated thoracic malignancies transcriptionally related to BAF‐deficient sarcomas. Nat Genet. 2015;47(10):1200‐1205. https://doi.org10.1038/ng.3399. 26343384 10.1038/ng.3399

[12] Hodges HC , Stanton BZ , Cermakova K , et al. Dominant‐negative SMARCA4 mutants alter the accessibility landscape of tissue‐unrestricted enhancers. Nat Struct Mol Biol. 2018;25(1):61‐72. https://doi.org10.1038/s41594‐017‐0007‐3. 29323272 10.1038/s41594-017-0007-3 PMC5909405

[13] Ganguly D , Sims M , Cai C , Fan M , Pfeffer LM . Chromatin remodeling factor BRG1 regulates stemness and chemosensitivity of glioma initiating cells. Stem Cells. 2018;36(12):1804‐1815. https://doi.org10.1002/stem.2909. 30171737 10.1002/stem.2909 PMC7427091

[14] Yang CH , Yue J , Pfeffer SR , et al. MicroRNA‐21 promotes glioblastoma tumorigenesis by down‐regulating insulin‐like growth factor‐binding protein‐3 (IGFBP3). J Biol Chem. 2014;289(36):25079‐25087. https://doi.org10.1074/jbc.M114.593863. 25059666 10.1074/jbc.M114.593863 PMC4155674

[15] Brennan CW , Verhaak RG , McKenna A , et al. The somatic genomic landscape of glioblastoma. Cell. 2013;155(2):462‐477. https://doi.org10.1016/j.cell.2013.09.034. 24120142 10.1016/j.cell.2013.09.034 PMC3910500

[16] Cancer Genome Atlas Research Network . Comprehensive genomic characterization defines human glioblastoma genes and core pathways. Nature. 2008;455(7216):1061‐1068. https://doi.org10.1038/nature07385 18772890 10.1038/nature07385 PMC2671642

[17] Zhao J , Chen AX , Gartrell RD , et al. Immune and genomic correlates of response to anti‐PD‐1 immunotherapy in glioblastoma. Nat Med. 2019;25(3):462‐469. https://doi.org10.1038/s41591‐019‐0349‐y. 30742119 10.1038/s41591-019-0349-y PMC6810613

[18] Cerami E , Gao J , Dogrusoz U , et al. The cBio cancer genomics portal: an open platform for exploring multidimensional cancer genomics data. Cancer Discov. 2012;2(5):401‐404. https://doi.org10.1158/2159‐8290.CD‐12‐0095. 22588877 10.1158/2159-8290.CD-12-0095 PMC3956037

[19] Bowman RL , Wang Q , Carro A , Verhaak RG , Squatrito M . GlioVis data portal for visualization and analysis of brain tumor expression datasets. Neuro Oncol. 2017;19(1):139‐141. https://doi.org10.1093/neuonc/now247. 28031383 10.1093/neuonc/now247 PMC5193031

[20] Yue J , Sheng Y , Ren A , Penmatsa S . A miR‐21 hairpin structure‐based gene knockdown vector. Biochem Biophys Res Commun. 2010;394(3):667‐672. https://doi.org10.1016/j.bbrc.2010.03.047. 20226761 10.1016/j.bbrc.2010.03.047 PMC2854175

[21] Yang CH , Fan M , Slominski AT , Yue J , Pfeffer LM . The role of constitutively activated STAT3 in B16 melanoma cells. Int J Interferon Cytokine Mediat Res. 2010;2010(2):1‐7. https://doi.org10.2147/IJICMR.S6657. 20814592 10.2147/IJICMR.S6657 PMC2931364

[22] Kadoch C , Crabtree GR . Mammalian SWI/SNF chromatin remodeling complexes and cancer: Mechanistic insights gained from human genomics. Sci Adv. 2015;1(5):e1500447. https://doi.org10.1126/sciadv.1500447 26601204 10.1126/sciadv.1500447 PMC4640607

[23] Verhaak RG , Hoadley KA , Purdom E , et al. Integrated genomic analysis identifies clinically relevant subtypes of glioblastoma characterized by abnormalities in PDGFRA, IDH1, EGFR, and NF1. Cancer Cell. 2010;17(1):98‐110. https://doi.org10.1016/j.ccr.2009.12.020. 20129251 10.1016/j.ccr.2009.12.020 PMC2818769

[24] Ganguly D , Cai C , Sims MM , et al. APELA expression in glioma, and its association with patient survival and tumor grade. Pharmaceuticals (Basel). 2019;12(1):45. https://doi.org10.3390/ph12010045 10.3390/ph12010045 PMC6469159 30917521

[25] Yang CH , Wang Y , Sims M , et al. MiRNA203 suppresses the expression of protumorigenic STAT1 in glioblastoma to inhibit tumorigenesis. Oncotarget. 2016;7(51):84017‐84029. https://doi.org10.18632/oncotarget.12401. 27705947 10.18632/oncotarget.12401 PMC5341291

[26] Ganguly D , Fan M , Yang CH , et al. The critical role that STAT3 plays in glioma‐initiating cells: STAT3 addiction in glioma. Oncotarget. 2018;9(31):22095‐22112. https://doi.org10.18632/oncotarget.25188. 29774125 10.18632/oncotarget.25188 PMC5955139

[27] Yang CH , Wang Y , Sims M , et al. MicroRNA203a suppresses glioma tumorigenesis through an ATM‐dependent interferon response pathway. Oncotarget. 2017;8(68):112980‐112991. https://doi.org10.18632/oncotarget.22945. 29348882 10.18632/oncotarget.22945 PMC5762567

[28] Yang CH , Wang Y , Sims M , Cai C , Pfeffer LM . MicroRNA‐1 suppresses glioblastoma in preclinical models by targeting fibronectin. Cancer Lett. 2019;465:59‐67. https://doi.org10.1016/j.canlet.2019.08.021. 31491450 10.1016/j.canlet.2019.08.021

[29] Bourgo RJ , Siddiqui H , Fox S , et al. SWI/SNF deficiency results in aberrant chromatin organization, mitotic failure, and diminished proliferative capacity. Mol Biol Cell. 2009;20(14):3192‐3199. https://doi.org10.1091/mbc.E08‐12‐1224. 19458193 10.1091/mbc.E08-12-1224 PMC2710832

[30] Flanagan JF , Peterson CL . A role for the yeast SWI/SNF complex in DNA replication. Nucleic Acids Res. 1999;27(9):2022‐2028. 10198436 10.1093/nar/27.9.2022 PMC148416

[31] Muchardt C , Yaniv M . ATP‐dependent chromatin remodelling: SWI/SNF and Co. are on the job. J Mol Biol. 1999;293(2):187‐198. 10.1006/jmbi.1999.2999 10529347

[32] Wilson BG , Roberts CW . SWI/SNF nucleosome remodellers and cancer. Nat Rev Cancer. 2011;11(7):481‐492. https://doi.org10.1038/nrc3068 21654818 10.1038/nrc3068

[33] Bultman SJ , Gebuhr TC , Magnuson T . A Brg1 mutation that uncouples ATPase activity from chromatin remodeling reveals an essential role for SWI/SNF‐related complexes in beta‐globin expression and erythroid development. Gene Dev. 2005;19(23):2849‐2861. 10.1101/gad.1364105 16287714 PMC1315392

[34] Ho L , Ronan JL , Wu J , et al. An embryonic stem cell chromatin remodeling complex, esBAF, is essential for embryonic stem cell self‐renewal and pluripotency. Proc Natl Acad Sci USA. 2009;106(13):5181‐5186. https://doi.org10.1073/pnas.0812889106. 19279220 10.1073/pnas.0812889106 PMC2654396

[35] Buscarlet M , Krasteva V , Ho L , et al. Essential role of BRG, the ATPase subunit of BAF chromatin remodeling complexes, in leukemia maintenance. Blood. 2014;123(11):1720‐1728. https://doi.org10.1182/blood‐2013‐02‐483495. 24478402 10.1182/blood-2013-02-483495 PMC3954053

[36] Lan J , Li H , Luo X , Hu J , Wang G . BRG1 promotes VEGF‐A expression and angiogenesis in human colorectal cancer cells. Exp Cell Res. 2017;360(2):236‐242. https://doi.org10.1016/j.yexcr.2017.09.013. 28899659 10.1016/j.yexcr.2017.09.013

[37] Liu X , Tian X , Wang F , Ma Y , Kornmann M , Yang Y . BRG1 promotes chemoresistance of pancreatic cancer cells through crosstalking with Akt signalling. Eur J Cancer. 2014;50(13):2251‐2262. https://doi.org10.1016/j.ejca.2014.05.017. 24953335 10.1016/j.ejca.2014.05.017

[38] Roy N , Malik S , Villanueva KE , et al. Brg1 promotes both tumor‐suppressive and oncogenic activities at distinct stages of pancreatic cancer formation. Genes Dev. 2015;29(6):658‐671. https://doi.org10.1101/gad.256628.114. 25792600 10.1101/gad.256628.114 PMC4378197

[39] Shi J , Whyte WA , Zepeda‐Mendoza CJ , et al. Role of SWI/SNF in acute leukemia maintenance and enhancer‐mediated Myc regulation. Genes Dev. 2013;27(24):2648‐2662. https://doi.org10.1101/gad.232710.113. 24285714 10.1101/gad.232710.113 PMC3877755

[40] Alhawiti NM , Al Mahri S , Aziz MA , Malik SS , Mohammad S . TXNIP in metabolic regulation: physiological role and therapeutic outlook. Curr Drug Targets. 2017;18(9):1095‐1103. https://doi.org10.2174/1389450118666170130145514. 28137209 10.2174/1389450118666170130145514 PMC5543564

[41] Li J , Yue Z , Xiong W , Sun P , You K , Wang J . TXNIP overexpression suppresses proliferation and induces apoptosis in SMMC7221 cells through ROS generation and MAPK pathway activation. Oncol Rep. 2017;37(6):3369‐3376. https://doi.org10.3892/or.2017.5577. 28440491 10.3892/or.2017.5577

[42] Malone CF , Emerson C , Ingraham R , et al. mTOR and HDAC inhibitors converge on the TXNIP/Thioredoxin pathway to cause catastrophic oxidative stress and regression of RAS‐driven tumors. Cancer Discov. 2017;7(12):1450‐1463. https://doi.org10.1158/2159‐8290.CD‐17‐0177. 28963352 10.1158/2159-8290.CD-17-0177 PMC5718976

[43] Nagaraj K , Lapkina‐Gendler L , Sarfstein R , et al. Identification of thioredoxin‐interacting protein (TXNIP) as a downstream target for IGF1 action. Proc Natl Acad Sci USA. 2018;115(5):1045‐1050. https://doi.org10.1073/pnas.1715930115. 29339473 10.1073/pnas.1715930115 PMC5798358

[44] Shen L , O'Shea JM , Kaadige MR , et al. Metabolic reprogramming in triple‐negative breast cancer through Myc suppression of TXNIP. Proc Natl Acad Sci USA. 2015;112(17):5425‐5430. https://doi.org10.1073/pnas.1501555112. 25870263 10.1073/pnas.1501555112 PMC4418882

[45] Wang H , Lathia JD , Wu Q , et al. Targeting interleukin 6 signaling suppresses glioma stem cell survival and tumor growth. Stem Cells. 2009;27(10):2393‐2404. https://doi.org10.1002/stem.188. 19658188 10.1002/stem.188 PMC2825688

[46] Tokunaga R , Zhang W , Naseem M , et al. CXCL9, CXCL10, CXCL11/CXCR3 axis for immune activation ‐ A target for novel cancer therapy. Cancer Treat Rev. 2018;63:40‐47. https://doi.org10.1016/j.ctrv.2017.11.007. 29207310 10.1016/j.ctrv.2017.11.007 PMC5801162

[47] Bidwell BN , Slaney CY , Withana NP , et al. Silencing of Irf7 pathways in breast cancer cells promotes bone metastasis through immune escape. Nat Med. 2012;18(8):1224‐1231. https://doi.org10.1038/nm.2830. 22820642 10.1038/nm.2830

[48] Kidder BL , Palmer S , Knott JG . SWI/SNF‐Brg1 regulates self‐renewal and occupies core pluripotency‐related genes in embryonic stem cells. Stem Cells. 2009;27(2):317‐328. https://doi.org10.1634/stemcells.2008‐0710. 19056910 10.1634/stemcells.2008-0710

[49] Zhang X , Li B , Li W , et al. Transcriptional repression by the BRG1‐SWI/SNF complex affects the pluripotency of human embryonic stem cells. Stem Cell Reports. 2014;3(3):460‐474. https://doi.org10.1016/j.stemcr.2014.07.004. 25241744 10.1016/j.stemcr.2014.07.004 PMC4266000

[50] Hiramatsu H , Kobayashi K , Kobayashi K , et al. The role of the SWI/SNF chromatin remodeling complex in maintaining the stemness of glioma initiating cells. Sci Rep. 2017;7(1):889. https://doi.org10.1038/s41598‐017‐00982‐3 28420882 10.1038/s41598-017-00982-3 PMC5429847

[51] Marquez‐Vilendrer SB , Rai SK , Gramling SJ , Lu L , Reisman DN . Loss of the SWI/SNF ATPase subunits BRM and BRG1 drives lung cancer development. Oncoscience. 2016;3(11–12):322‐336. https://doi.org10.18632/oncoscience.323. 28105457 10.18632/oncoscience.323 PMC5235921

[52] Hendricks KB , Shanahan F , Lees E . Role for BRG1 in cell cycle control and tumor suppression. Mol Cell Biol. 2004;24(1):362‐376. 14673169 10.1128/MCB.24.1.362-376.2004 PMC303366

[53] Dunaief JL , Strober BE , Guha S , et al. The retinoblastoma protein and BRG1 form a complex and cooperate to induce cell cycle arrest. Cell. 1994;79(1):119‐130. 7923370 10.1016/0092-8674(94)90405-7

[54] Shanahan F , Seghezzi W , Parry D , Mahony D , Lees E . Cyclin E associates with BAF155 and BRG1, components of the mammalian SWI‐SNF complex, and alters the ability of BRG1 to induce growth arrest. Mol Cell Biol. 1999;19(2):1460‐1469. 9891079 10.1128/mcb.19.2.1460 PMC116074

[55] Bao S , Wu Q , McLendon RE , et al. Glioma stem cells promote radioresistance by preferential activation of the DNA damage response. Nature. 2006;444(7120):756‐760. https://doi.org10.1038/nature05236. 17051156 10.1038/nature05236

[56] Deshmukh A , Deshpande K , Arfuso F , Newsholme P , Dharmarajan A . Cancer stem cell metabolism: a potential target for cancer therapy. Mol Cancer. 2016;15(1):69. https://doi.org10.1186/s12943‐016‐0555‐x 27825361 10.1186/s12943-016-0555-x PMC5101698

[57] Kothandapani A , Gopalakrishnan K , Kahali B , Reisman D , Patrick SM . Downregulation of SWI/SNF chromatin remodeling factor subunits modulates cisplatin cytotoxicity. Exp Cell Res. 2012;318(16):1973‐1986. https://doi.org10.1016/j.yexcr.2012.06.011. 22721696 10.1016/j.yexcr.2012.06.011 PMC3408789

[58] Qi W , Wang R , Chen H , et al. BRG1 promotes the repair of DNA double‐strand breaks by facilitating the replacement of RPA with RAD51. J Cell Sci. 2015;128(2):317‐330. https://doi.org10.1242/jcs.159103. 25395584 10.1242/jcs.159103 PMC4294775

